# Local anesthetics systemic toxicity in children: analysis of the French pharmacovigilance database

**DOI:** 10.1186/s12887-023-04126-7

**Published:** 2023-06-24

**Authors:** Arnaud Schweitzer-Chaput, Delphine Callot, Naim Bouazza, Fabrice Lesage, Mehdi Oualha, Nathalie Paret, Marie Boyer-Gervoise, Jean-Marc Treluyer, Laurent Chouchana

**Affiliations:** 1 Centre Régional de Pharmacovigilance, Service de Pharmacologie, Hôpital Cochin, AP-HP, Paris, France; 2 Pharmacologie Et Évaluation Des Thérapeutiques Chez L’enfant Et La Femme Enceinte (EA 7323), Université de Paris, Paris, France; 3 Unité de Recherche Clinique Paris Descartes Necker Cochin, AP-HP, Paris, France; 4 Service de Réanimation Pédiatrique, Hôpital Necker, Paris, France; 5 Centre Antipoison, Hospices Civils De Lyon, Lyon, France; 6 Centre Régional de Pharmacovigilance, Service de Pharmacologie Clinique, Assistance Publique Hôpitaux de Marseille, Marseille, France

**Keywords:** Local anesthetics, Pharmacovigilance, Pediatric, Systemic toxicity, Adverse drug reaction

## Abstract

**Purpose:**

To characterize clinical profile of pediatric local anesthetic (LA) systemic toxicity (LAST) and to identify determinants of life-threatening outcomes.

**Methods:**

Spontaneous reports notified to the French Pharmacovigilance Network were retrieved and followed by a case-by-case review, according to the following criteria: LA as suspected drug, age < 18 years, adverse drug reactions related to nervous system, cardiac, respiratory, psychiatric or general disorders. Multivariate logistic regression analysis was performed to identify factors leading to life-threatening reaction (i.e. continuous seizures or cardiorespiratory arrest).

**Results:**

Among 512 cases retrieved, 64 LAST cases were included (neonates 11%, infants 30%, children 36%, adolescents 23%) mainly involving lidocaine (47%), lidocaine + prilocaine (22%) and ropivacaine (14%). Toxicity profiles were neurological (58%), cardiac (11%) or mixed (20%) and 7 patients (11%) developed methemoglobinemia. LAST was life-threatening for 23 patients (36%) and 2 patients died. Doses were above recommendations in 26 patients (41%) and were not different between life-threatening and non-life-threatening cases. The context of use (general and orthopedic surgery, *p* = 0.006) and the type of LA agent (lidocaine, *p* = 0.016) were independently associated with a life-threatening outcome.

**Conclusion:**

In this national retrospective analysis, LAST in children appear to be a rare event. Neurological and cardiac signs were the most frequently reported reactions. LAST in children can be life-threatening, even at therapeutic doses. Although a fatal outcome may anecdotally occur, the vast majority of patients recovered after appropriate medical care.

**Supplementary Information:**

The online version contains supplementary material available at 10.1186/s12887-023-04126-7.

## What is already known on this topic?

Local anesthetics can cause severe systemic toxicity after intravenous absorption from injection site (topical, or subcutaneous), particularly in the pediatric population. However, the factors associated with these systemic reactions are poorly known.

## What is new?

Local anesthetics systemic toxicity can lead to severe systemic reactions, up to death. The majority of these reactions occurred within one hour of administration, some occurred several hours later. Doses used were below or above the maximum recommended doses. Children should be carefully monitored at least during one hour after LA administration. The use of recommended doses does not prevent to onset of systemic toxicity. Lipid emulsion was administered in very few cases, which may underline a lack of awareness of its indication as an antidote in case of systemic toxicity. In case of transdermal application in ambulatory settings, particular attention should be drawn to ensure that parents appropriately follow the prescription of local anaesthetics.

## Introduction

Local anesthetics (LA) are used in several medical conditions, from stitches to surgeries. Since the first use of cocaine for eye surgery in 1884, several molecules have been synthesized the most common being lidocaine [1]. LA bind to voltage-gated sodium channels of nerve cells, preventing the entry of sodium ions. This results in further blocking nerve depolarization and therefore pain transmission [2]. According to their administration site, LA are intended to be used for a local or regional action (topical or subcutaneous administration), or for epidural or spinal anesthesia.

LA are drugs having an overall very good safety profile. In some cases, they are associated with benign injection site reactions, such as rash. However, severe adverse drug reactions (ADRs) may occur after systemic absorption or inadvertent intravascular injection [3, 4]. Systemic toxicity of LA being concentration-dependent, characteristics of the injection site as well as administration method play a crucial role in their absorption and transfer to systematic blood circulation [5]. Local anesthetics systemic toxicity (LAST) is characterized by neurological and cardio-pulmonary adverse drug reactions that may be serious and even life-threatening [6, 7]. Early management, and if necessary administration of a non-specific antidote such as lipid emulsion, leads to favorable outcome in most cases [8, 9].

To date, LAST cases have been reported in adults and children after local or regional uses [10–13]. However, in relation with specific pharmacokinetics characteristics, LAST in children may be different from adults and toxic doses or clinical outcome are poorly known [14, 15].

In this study, we aimed to describe the characteristics of the LAST pediatric cases reported to the French Pharmacovigilance Network and to identify determinants associated with life-threatening consequences.

## Materials and methods

### Data source

This is a retrospective observational study of spontaneous reports of LAST cases reported to the French Pharmacovigilance Network. Cases were spontaneously reported by healthcare workers or patients. They were further registered anonymously in the French Pharmacovigilance Database after medical assessment by clinical pharmacologists from the regional centers of pharmacovigilance across the country.

### Data extraction

Cases reported from database inception in 1986 until February 2019, 28th, in patients under 18 years and with the following suspected drugs were retrieved: lidocaine, procaine, chloroprocaine, prilocaine, mepivacaine, bupivacaine, levobupivacaine or ropivacaine. In order to identify cases of LAST, the Medical Dictionary for Drug Regulatory Activities (MedDRA) was used [16]. Cases with reactions related to the following System Organ Classes (SOCs) from MedDRA were extracted: cardiac disorders, general disorders and administration site conditions, nervous system disorders, psychiatric disorders and respiratory, thoracic and mediastinal disorders.

### Data analysis

A case-by-case review by a clinical pharmacologist was performed to collect variables characterizing the patient, the situation of occurrence, the reactions and its management. Doubtful cases were further reviewed by a senior clinical pharmacologist. Exclusion criteria were: LA use as excipient (e.g.: for intramuscular route), local reaction only (e.g.: rash, skin reaction), reaction unrelated to LAST (e.g.: ineffectiveness), another diagnosis appearing more likely (e.g.: timing of events poorly compatible, other likely suspect drugs), vaccination-related malaise, maternal exposure to LA (epidural during delivery), non-compatible time to onset and insufficient data.

Seriousness of cases was defined, according to the WHO, as the occurrence of death, life-threatening adverse event, inpatient hospitalization or prolongation of an existing hospitalization, significant disability or requirement of intervention to prevent any of these [17]. Life-threatening cases were defined by the occurrence of at least a neurological or cardiorespiratory ADRs: seizures, cardiac or cardiorespiratory arrest.

Compliance with the recommended doses for each case was analyzed using the French summary of product characteristics.

The place of care of the LAST cases was analyzed only in the cases in which the first signs and symptoms of LA intoxication appeared in a non-hospital setting.

### Statistical analysis

The data are presented as proportions for categorical data and as median and interquartile range (IQR) for quantitative data. Quantitative variables were compared in non-parametric Wilcoxon tests and proportions were compared in Fisher’s exact tests or chi-squared tests, as appropriate. A multivariable logistic regression analysis, including variables not related to the outcome (i.e. occurrence of a life-threatening condition), was performed. The selection of the final variables retained in the final multivariable model was achieved by bidirectional stepwise using Akaike’s information criterion (AIC). Analysis was performed using R.

This study, analyzing anonymous data, being retrospective and non-interventional, the approval of an Ethics committee was not necessary according to French laws [18].

## Results

### LAST characteristics

**Fig. 1:**
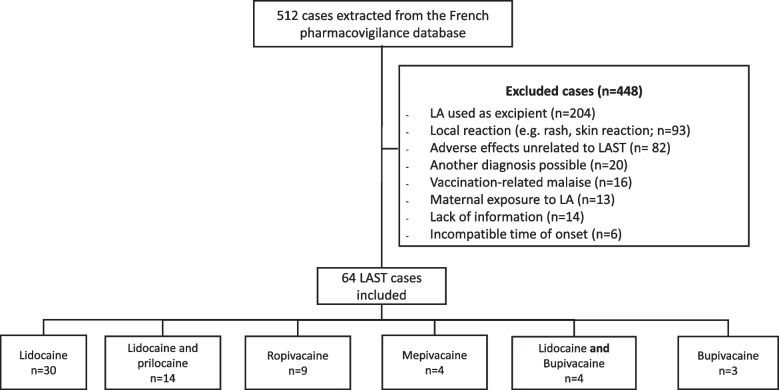
Flowchart of local anesthetics systemic toxicity cases included in the study

A total of 512 cases were extracted from the French Pharmacovigilance Database. After case-by-case review, 447 cases were excluded, as shown in Fig. 1. Cases characteristics are presented in Table 1, Supplementary Table 1 and Supplementary Figure 1. Among the 64 cases included in this study, 30 were reported with lidocaine, 14 with lidocaine and prilocaine, 9 with ropivacaine, 4 with mepivacaine, 4 with lidocaine and bupivacaine and 3 with bupivacaine.

**Table 1:** Characteristics of the local anesthesic systemic toxicity cases

	Lidocaine (*n* = 30)	Lidocaine + prilocaine (*n* = 14)	Ropivacaine (*n* = 9)	Mepivacaine (*n* = 4)	Lidocaine + bupivacaine (*n* = 4)	Bupivacaine (*n* = 3)	Overall (*n* = 64)
Sex, n (%)							
Boy	20 (66.7)	4 (28.6)	5 (55.6)	3 (75)	4 (100)	2 (66.7)	38 (59.4)
Age, n (%)							
Newborn (0 – 28 days)	4 (13.3)	1 (7.1)	1 (11.1)	-	-	1 (33.3)	7 (10.9)
Infant (29 days – 2 years)	12 (40)	4 (28.6)	2 (22.2)	-	1 (25)	-	19 (29.7)
Child (2 – 11 years)	8 (26.7)	8 (57.1)	3 (33.3)	-	3 (75)	1 (33.3)	23 (35.9)
Adolescent (12 – 18 years)	6 (20)	1 (7.1)	3 (33.3)	4 (100)	-	1 (33.3)	15 (23.4)
Settings at adverse reaction onset, n (%)							
Hospital	11 (36.7)	-	9 (100)	2 (50)	4 (100)	3 (100)	29 (45.3)
General practitioner office	6 (20)	11 (78.6)	-	-	-	-	17 (26.6)
Private clinic	8 (26.7)	-	-	-	-	-	8 (12.5)
Dental office	4 (13.3)	-	-	2 (50)	-	-	6 (9.4)
Home	1 (3.3)	3 (21.4)	-	-	-	-	4 (6.3)
Context of use, n (%)							
General and orthopedic surgery	2 (6.7)	-	9 (100)	-	3 (75)	2 (66.7)	16 (25)
Small act of surgery or diagnosis	8 (26.7)	2 (14.3)	-	1 (25)	1 (25)	-	12 (18.8)
Posthectomy	10 (33.3)	-	-	-	-	-	10 (15.6)
Dental Surgery	5 (16.7)	-	-	3 (75)	-	-	8 (12.5)
Cutaneous excision	2 (6.7)	6 (42.9)	-	-	-	-	8 (12.5)
Vaccination	-	6 (42.9)	-	-	-	-	6 (9.4)
Tonsillectomy	2 (6.7)	-	-	-	-	1 (33.3)	3 (4.7)
Involuntary ingestion	1 (3.3)	-	-	-	-	-	1 (1.6)
Route of administration, n (%)							
Local–regional and nerve block	23 (76.7)	-	4 (44.4)	1 (25)	2 (50)	2 (66.7)	32 (50)
Topical (transdermal, mucosal application)	3 (10)	14 (100)	-	-	-	-	17 (26.6)
Epidural	-	-	5 (55.6)	-	2 (50)	1 (33.3)	8 (12.5)
Dental	3 (10)	-	-	3 (75)	-	-	6 (9.3)
Other	1 (3.3)^a^						1 (1.6)
Compliance with recommended doses, n (%)							
Above maximum recommended doses	10 (33.3)	10 (71.4)	3 (33.3)	1 (25)	1 (25)	1 (33.3)	26 (40.6)
*Intentional*	*9 (30)*	*9 (64.3)*	*1 (11.1)*	*1 (25)*	*1 (25)*	*1 (33.3)*	*22 (34.4)*
*Unintentional in the context of medical error*	*1 (3.3)*	*1 (7)*	*2 (22.2)*	*-*	*-*	*-*	*4 (6.3)*
Below the maximum recommended dose	12 (40)	4 (44.4)	5 (55.6)	3 (75)	-	-	24 (37.5)
Dose used not known or no recommendation	8 (26.7)	-	1 (11.1)	-	3 (75)	2 (66.7)	14 (21.9)
Adverse reactions, n (%)							
Neurological	44 (61.1)	21 (56.8)	10 (43.4)	10 (91)	2 (50)	3 (25)	90 (56.6)
Cardiorespiratory	28 (38.9)	9 (24.3)	13 (65.5)	1 (9.1)	2 (50)	9 (75)	62 (39)
Methemoglobinemia	-	7 (18.9)	-	-	-	-	7 (4.4)
Seriousness, n (%)							
Yes^b^	27 (90)	12 (85.7)	9 (100)	4 (100)	2 (50)	3 (100)	57 (89.1)
Severity, n (%)							
Life-threatening	15 (65.2)	-	6 (26.1)	1 (4.3)	-	1 (4.3)	23 (36)
Time to onset							
Median [IQR] in min	15 [3-30]	60 [5–150]	300 [5–540]	20 [12–295]	-^c^	195 [113–278]	20 [5–113]
Range							
< 10 min, n (%)	9 (30)	2 (14.3)	3 (33.3)	1 (25)	3 (75)	-	18 (28.1)
10–60 min, n (%)	13 (43.3)	4 (28.6)	1 (11.1)	1 (25)	1 (25)	2 (66.7)	22 (34.4)
1–12 h, n (%)	2 (6.7)	4 (28.6)	3 (33.3)	1 (25)	-	1 (33.3)	11 (17.2)
> 12 h, n (%)	-	1 (7.1)	2 (22.2)	-	-	-	3 (4.7)
Unknown, n (%)	6 (20)	3 (21.4)	-	1 (25)	-	-	10 (15.6)
Place of care^d^, n (%)							
Conventional hospitalization	6 (30)	10 (71.4)	-	1 (50)	-	-	33 (51.6)
Intensive care unit	11 (55)	1 (7.1)	-	-	-	-	18 (28.1)
Hospitalization unspecified	-	2 (14.3)	-	1 (50)	-	-	9 (14.1)
No hospitalization	3 (15)	1 (7.1)	-	-	-	-	4 (6.25)
Therapeutic management, n (%)							
Symptomatic treatment	20 (66.7)	3 (21.4)	6 (66.7)	-	2 (50)	3 (100)	34 (53.1)
Simple monitoring	6 (20)	9 (64.3)	1 (11.1)	2 (50)	2 (50)	-	20 (31.3)
Unknown	3 (10)	2 (18.2)	-	1 (25)	-	-	6 (9.4)
Lipid emulsion	1 (3.3)	-	2 (22.2)	1 (25)	-	-	4 (6.3)
Final outcome, n (%)							
Recovery	28 (93.3)	14 (100)	9 (100)	4 (100)	4 (100)	3 (100)	62 (96.9)
Death	2 (6.7)	-	-	-	-	-	2 (3.1)

*IQR* interquartile range

^a^One case of involuntary ingestion

^b^Seriousness of cases was defined, according to the WHO, as the occurrence of death, life-threatening adverse event, inpatient hospitalization or prolongation of an existing hospitalization, significant disability or requirement of intervention to prevent any of these

^c^no exact timing reported

^d^only cases where symptoms of intoxication first appeared in an out-of-hospital setting were included

Overall, cases mainly concerned child between 2 and 11 years, mostly boys and occurred essentially in hospitals after a loco-regional nerve block. Most cases followed a small act of surgery (such as stitches, fibroscopy or cutaneous biopsy) or general and orthopedic surgery. We observed a wide variability in administered doses as in 26 (41%) and 24 (38%) cases, doses were above and below the maximum recommended doses, respectively. LA plasma level was reported in 15 cases (Supplementary Table 2). In all but one case, plasma assay was performed at least one hour after the onset of symptoms and was thus uninterpretable. For the case in which plasma level was assayed at the time of the convulsions, it was above the toxicity threshold.

### Life-threatening condition

A life-threatening condition was observed in 23 (36%) cases (Table 1). Most of them concerned boys from 29 days to 2 years of age, occurred mostly in hospital following a loco-regional nerve block or a general or orthopedic surgery. Compliance with recommended doses did not differ between life-threatening and non-life-threatening cases (Supplementary Table 1). The majority of non-life-threatening cases were managed in conventional hospitalization mainly with simple monitoring or symptomatic treatment (such as CPR, oxygenation and mostly benzodiazepine) whereas life-threatening cases required symptomatic treatment or non-specific antidote.

**Fig. 2:**
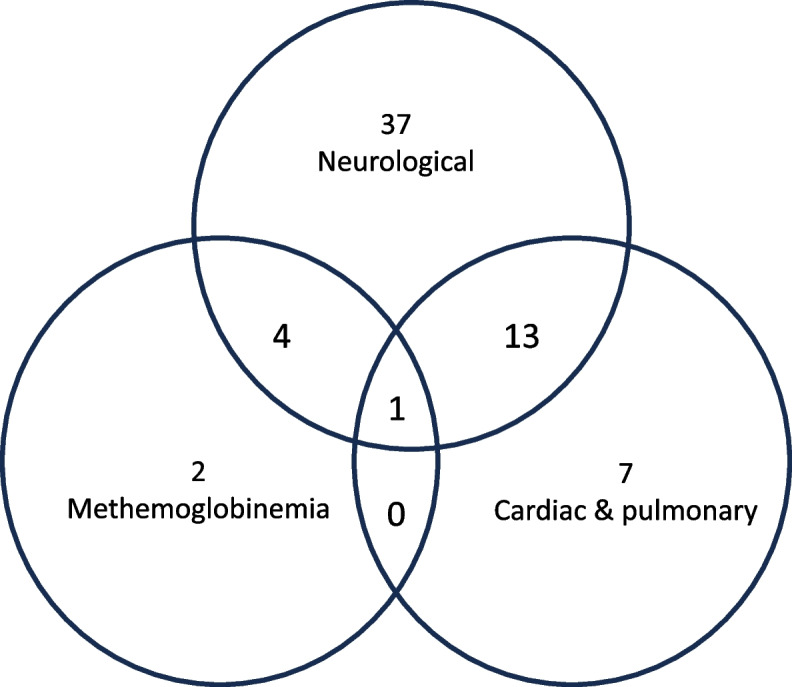
Adverse drug reaction pattern of local anesthetics systemic toxicity cases in children

In the vast majority of LAST cases occurred less than 10 min or between 10 to 60 min after LA administration, this being not different between life-threatening and non-life-threatening cases (Supplementary Table 3). Reactions were mostly neurological, with a majority of malaise and single convulsion for non-life-threatening cases and seizures for life threatening cases (Tables 1 and 2, Fig. 2 and Supplementary Table 3). Overall, few prodromal signs were described before the major clinical signs, including in life-threatening cases. Seven cases of methemoglobinemia following the use of lidocaine and prilocaine were reported. Those cases concerned 4 infants and 3 children, with no life-threatening situations. These cases occurred as a result of parents administering excessive doses of EMLA® creams or patches, due to poor or no explanation to parents prior to administration. In addition, no cluster by year was identified.

**Table 2:** Details of reported adverse reactions

	Lidocaine	Lidocaine + prilocaine	Ropivacaine	Mepivacaine	Lidocaine + bupivacaine	Bupivacaine	Overall
Neurological, n (%)	**44 (61.1)**	**21 (56.8)**	**10 (43.4)**	**10 (91)**	**2 (50)**	**3 (25)**	**90 (56.6)**
Unique convulsion	12 (16.7)	5 (13.5)	-	1 (9.1)	2 (50)	-	20 (12.6)
Seizures	12 (16.7)	-	5 (21.8)	1 (9.1)	-	-	18 (11.3)
Malaise	6 (8.3)	4 (10.8)	-	2 (18.2)	-	1 (8.3)	13 (8.2)
Sleepiness	2 (2.8)	7 (18.9)	1 (4.3)	2 (18.2)	-	-	12 (7.5)
Hypotonia	3 (4.2)	3 (8.1)	1 (4.3)	-	-	1 (8.3)	8 (5)
Abnormal movements	2 (2.8)	1 (2.7)	1 (4.3)	-	-	1 (8.3)	5 (3.1)
Loss of consciousness	2 (2.8)	-	-	1 (9.1)	-	-	3 (1.9)
Coma	3 (4.2)	-	-	-	-	-	3 (1.9)
Cries	2 (2.8)	-	-	-	-	-	2 (1.3)
Other	-	1 (2.7)^a^	2 (8.6)^b^	3 (27.3)^c^	-	-	6 (3.8)
Cardiorespiratory, n (%)	**28 (38.9)**	**9 (24.3)**	**13 (65.5)**	**1 (9.1)**	**2 (50)**	**9 (75)**	**62 (39)**
Cyanosis	6 (8.3)	7 (18.9)	1 (4.3)	-	-	-	14 (8.8)
Bradycardia	5 (6.9)	1 (2.7)	4 (17.4)	1 (9.1)	-	2 (16.7)	13 (8.2)
Hypoxia	7 (9.7)	1 (2.7)	3 (13)	-	-	1 (8.3)	12 (7.5)
Cardiorespiratory arrest	4 (5.6)	-	1 (4.3)	-	-	1 (8.3)	6 (3.8)
Conduction disorders	-	-	2 (8.7)	-	2 (50)	1 (8.3)	5 (3.1)
Tachycardia	3 (4.2)	-	1 (4.3)	-	-	1 (8.3)	5 (3.1)
Hypotension	1 (1.4)	-	-	-	-	2 (16.7)	3 (1.9)
Respiratory distress	2 (2.8)	-	-	-	-	1 (8.3)	3 (1.9)
Cardiac arrest	-	-	1 (4.3)	-	-	-	1 (0.6)
Methemoglobinemia, n (%)	**-**	**7 (18.9)**	**-**	**-**	**-**	**-**	**7 (4.4)**

^a^Confusion

^b^Dysarthria and confusions

^c^Dysarthria, vertigo and memory disorders

The univariate analysis showed that the route of administration (*p* = 0.004), the context of use (*p* = 0.02) and the type of drug (*p* = 0.0008) were significantly associated with the occurrence of a life-threatening condition, whereas patient age, patient sex, local anesthetic dosage were not (Supplementary Table 1). Adjusted multivariate analysis showed that only the context of use (general and orthopedic surgery, adjusted OR [95% CI], 22.7 [2.5–205.9]) and the type of local anesthetics (lidocaine, adjusted OR [95% CI]), 14.3 [1.6–125.9]) remained significant.

### Use of specific non-specific antidote

The non-specific antidote, lipid emulsion, was used in 4 cases (age between 4 months and 18 years), 3 of them after a life-threatening adverse reaction occurred.

For instance, a 4 months child received lidocaine, at a dosage of 16 mg/kg, for a posthectomy in a general practitioner office. Twenty minutes after administration, a seizure occurred. Convulsions rapidly ceased under intrarectal benzodiazepine, but were followed by a cardiorespiratory arrest requiring cardiopulmonary resuscitation (CPR). The newborn was transferred into an intensive care unit with a saturation of 45%, a ventricular tachycardia with rapid return to sinus rhythm, where lipid emulsion was administered and the patient intubated. Another cardiorespiratory arrest occurred the next day following extubation. Finally, no long-term complications were reported. Plasma level of lidocaine was measured at 3 h after lidocaine administration resulting in a plasma level 4 mg/L (therapeutic level < 5 mg/L).

In another case, 30 to 40 mL of 2% mepivacaine and 2 mg of midazolam were used for loco-regional anesthesia for a transposition of the ulnar nerve of the left elbow on an 18 years old patient. The patient was discharged after the operation. Approximately 10 h after administration of LA, the patient went to the emergency department after the occurrence of vertigo, dysarthria and memory disorders. At admission, the patient presents with normal blood pressure and heart rate, a Glasgow coma scale of 15, intermittent dysarthria and anterograde amnesia. Symptoms improved one hour after administration of a lipid emulsion and completely recovered the next day.

In two cases, lipid emulsion was used after the administration of ropivacaine, following an administration with the use of an overly concentrated form of ropivacaine. In the first case, an ilio-fascial block was performed on 3 years old child after a femur fracture. A 7.5 mg/mL solution was mistakenly used instead of a 2 mg/mL solution, resulting in the administration of 112.5 mg of ropivacaine instead of 30 mg. Seizures occurred 5 min after injection, treated by intrarectal benzodiazepine and intravenous lipid emulsion. Convulsions stopped after benzodiazepine administration, and no cardiac signs or further adverse reaction occurred. Plasma level of ropivacaine was performed 1.5 h after administration, resulting in 1.74 mg/L. The second case concerned a 10 months old child, for whom an ilio-fascial block was performed before placing a closed femur fracture under traction. By mistake, a 10 mg/mL solution of ropivacaine instead of 2 mg/mL was used, resulting in the administration of 75 mg of ropivacaine. Approximately 5 min after administration, seizures occurred. Lipid emulsion and benzodiazepine were immediately administered, stopping the seizures, and no cardiac signs or further ADRs occurred.

### Clinical outcome

Sixty-two patients recovered and two died (3.2%).. In one case, a 19-month-old infant accidentally swallowed the content of a 2% lidocaine oral solution. The newborn quickly became hypotonic and cyanotic. After the arrival of the rescue, the newborn presented a bradycardia and seizures. Benzodiazepine administration and CPR were performed but were unsuccessful. The second case concerned a one-month-old newborn suffering from cystic fibrosis in whom lidocaine has been used for visceral surgery. Long-lasting seizures occurred without other detailed mentioned. Plasma level of lidocaine was performed 24 h after administration reaching 1.3 mg/L. Several days after the event, the newborn presented a coma, and a cerebral echography showed a severe cerebral ischemia. The newborn died approximately 2 weeks after the event.

## Discussion

To our knowledge, we report here one of the largest retrospective series of 64 LAST cases in children, at a national level [13]. Neurological reactions were the most frequently reported, followed by combined neurological and cardiac reactions, in line with the literature [11, 13, 19, 20]. All patients recovered except two infants with fatal outcome. Our multivariate model showed that the use of lidocaine, and a context of general and orthopedic surgery were significantly associated with the occurrence of a life-threatening reaction. However, interestingly neither patient age nor patient sex, nor local anesthetic dosages were significantly associated with the occurrence of a life-threatening condition.

LAST characteristics are related to neurological and cardio-pulmonary injuries. The first symptoms described in the literature are usually neurological, including prodromal symptoms like dizziness, confusion, dysarthria, tinnitus and metallic taste, followed by seizures up to seizures with loss of consciousness [21]. They are the result of active diffusion, involving a proton-antiporter flux as well as passive diffusion [22]. As dose increase, cardiovascular toxicity appears with symptoms like bradycardia, hypotension, ST-segment changes, arrhythmia or tachycardia that may lead to cardiorespiratory arrest. LAST diagnosis is based on clinical symptoms. Given the profile of this rapidly evolving intoxication, LA plasma assay is rarely performed during the first moments in clinical practice. Hence, as illustrated by the cases described here, plasma level is generally poorly informative.

To date, most reports have been related to long acting LA such as bupivacaine [13]. In our retrospective study, lidocaine was the most frequently involved in pediatric LAST cases. Although it is a short-acting LA, theoretically leading to less risk of systemic intoxication compared to long-acting LA, lidocaine is probably the most commonly used LA in children [23]. It is available in a wide variety of dosage forms (injectable, patch, gel for skin application, spray, etc.). Furthermore, it is used in children in a wide variety of clinical situations by pediatricians and is not restricted to anesthesiologists as are bupivacaine or ropivacaine. Hence, this common use associated with a trivialization of the dose limits and administration precautions may increase the number of LAST cases overall.

In the literature, symptoms of intoxication are generally described as occurring within a few minutes following administration. Some authors even describe cases occurring more than five minutes after administration as "atypical" [11]. However, in our study, a majority of cases occurred 10 to 60 min after administration, and sometimes later, as described in previous studies [20]. This underlines the need to closely monitor patients during at least one hour following the administration of LA. In children, this monitoring may for instance imply the parents. Indeed, the majority of the reactions being non-specific, the parents are probably the most likely to detect an unusual behavior of their child. However, this involvement requires an appropriate explanation of the potential risks by healthcare professional without creating excessive anxiety. In addition, most of the warning minor signs commonly described in LA overdose (metallic taste, tinnitus, paresthesia…) were not found in our cases series. This may be related with a lack of verbalization and/or of knowledge of these sensations by infants or children. Indeed, particularly in newborns and young children, warning signs consist mainly of crying, which is not specific enough to alert to potential toxicity. One could also hypothesize that there may be fewer warning signs in the pediatric population, compared to adults, preventing healthcare provider from early detection of a LAST. Moreover, our cases series based on reports from the French pharmacovigilance network are mostly serious. Therefore, minor signs may not be clearly mentioned in the case commentary.

Our findings as previous cases found that LAST cases were disproportionately reported in infants. For instance, infants and children show a higher total body-surface area to body mass ratio than adults, resulting in a higher exposure to topical LA [24]. In terms of distribution, lower quantity of total plasma protein is seen in the neonate and young infant, especially alpha-1-acid glycoprotein, the major binding protein for LA, therefore increasing the free concentration of drugs [25]. Concerning drug metabolism, drug-metabolizing enzyme levels like cytochrome P450 gradually increase during childhood. Finally, concerning elimination there is a maturation of the renal function in a dynamic way, starting from fetal life to early childhood. This development is related with an increase in the glomerular filtration rate during childhood, and thus may altered the plasma level of drugs [24]. In line with these pediatric characteristics, reduced pediatric doses have been proposed [26]. However, our findings highlight that about half of the LAST cases occurred with the use of recommended doses, as shown by other studies [13, 20]. This observation emphasizes the fact that even when using recommended doses, vigilance must be maintained for early identification of LAST and an appropriate administration technique must be used, especially when administered by injection (subcutaneous or epidural) [27].

LAST management is essentially based on symptomatic cares. It includes the discontinuation of administration of LA, the use of an anti-seizure agent (usually benzodiazepine) and management of cardiac reactions if necessary [6, 28]. In life-threatening conditions, 20% lipid emulsion is recommended [8, 29]. The mechanism of lipid emulsion is not fully understood, and several theories have been proposed [30]. Lipid emulsion could work as a “lipid sink”, by forming a lipid phase in the plasma and attract free lipophilic drugs like LA from the plasma. Efficiency of lipid emulsion could also have a metabolic origin, by acting as fatty acid substrate and increasing the production of adenosine triphosphate by mitochondria in the heart. In addition, triglycerides contained in the lipid emulsion could activate myocardial calcium and potassium channels to increase cardiac function [8, 31]. The use of this lipid emulsion in LAST management has been recommended by the American Society of Regional Anesthesia and Pain Medicine (ASRA) in 2010 [32]. In this case series over 40 years, of 11 life-threatening cases occurring after those recommendations, lipid emulsion have been administered in 4 cases (36%). Consistently, a recent literature review found a similar rate of lipid emulsion use overall [13]. This under-use may be related to a lack of knowledge of the recommendations for the management of these poisonings [19, 20]. Furthermore, in order to ensure the earliest possible use in the diagnosis of intoxication,, efforts must be performed for lipid emulsion availability in all clinical departments using LA.

Finally, several cases involved patches or cream containing lidocaine and prilocaine, mainly prior a vaccination or minor skin surgery. LAST was a consequence of the application of whole tubes (up to 8) used for dermatological biopsies, of whole patches in newborns, or of patches applied during a whole night on a newborn. In addition to the risk of LAST, the misuse of eutectic mixture of lidocaine and prilocaine can lead to methemoglobinemia (MetHb). MetHb is the oxidized form of hemoglobin, when the iron moiety is oxidized from its ferrous state Fe^2+^ to its ferric state Fe^3+^. This oxidized MetHb has a poor oxygen affinity reducing the oxygen delivery to tissue leading to hypoxia. When massively exposed to an exogenous oxidizing agent, such as chemicals (pesticides, nitrates…) or drugs (LA, dapsone, chloroquine…) protective pathways are overwhelmed leading to symptoms of hypoxia and requiring the administration of an antidote such as methylene blue [33]. Among LA, MetHb is a specific side effect of the use of prilocaine due to their oxidative properties [34], reported here in 7 cases (Table 1) and in several case reports in the literature [10]. These cases show the need to not trivialize the use of these drugs, particularly in outpatient settings where post-procedure monitoring will not be optimal, and where crying will be the main warning sign. Furthermore, newborns exhibit thinner and more permeable skin, increasing the risk of systemic reactions and requiring extra caution.

### Limitations

The most important limitation of our study relies on the nature of the pharmacovigilance database. Inherent to spontaneous reporting, underreporting is estimated at around 90% [35]. Furthermore, serious cases are more likely to be reported than mild or minor cases resulting in a possible reporting bias. However, although incidence cannot be assessed, LAST in children appear to be very rare on this national series over more than 30 years. Furthermore, this database allowed the assessment of this issue at a national level. In addition, the data used in this study are collected mainly from case narratives. This is a descriptive text of the events and chronology of the case, according to the medical record analyzed by clinical pharmacologists. These sources of information are inherently heterogeneous. Finally, regarding the statistical analysis, the very wide confidence intervals suggest the presence of confounding factors and selection biases due to the retrospective design.

## Conclusion

LAST in children appear to be a rare event according to the relatively low number of cases reported over more than 30 years at a national level. The pediatric population has specific pharmacokinetic characteristics, evolving with age, making it at higher risk, particularly in infants [36, 37]. It can lead to severe ADRs in more than a third of cases life-threatening. LAST occur in majority less than one hour after LA administration. Early management is essential, and requires specialized cares, including intravenous lipid emulsion in life threatening cases. Finally, the large number of cases that occurred in the absence of identified overdosage emphasizes the need to respect the administration procedures and patient monitoring. In ambulatory settings, the correct explanation and understanding of the prescription is essential, as well as the caution to not trivialize the use of LA.

## Supplementary Information

**Additional file 1:** **Supplementary Table 1.** Clinical presentation and univariate statistical analysis of life-threatening and non-life-threatening cases.

**Additional file 2:** **Supplementary Table 2.** Description of cases reporting plasma level of local anesthetic.

**Additional file 3:** **Supplementary Table 3.** Description of adverse effects and management of life-threatening and non-life-threatening cases.

**Additional file 4:** **Supplementary Figure 1.** Number of reported LAST cases by molecule.

## Data Availability

The datasets used and/or analyzed during the current study are available from the corresponding author on reasonable request.

## Abbreviations

ADRs: Adverse drug reactions
AIC: Akaike’s information criterion
ASRA: American Society of Regional Anesthesia and Pain Medicine
CPR: Cardiopulmonary resuscitation
IQR: Interquartile range
LA: Local anesthetics
LAST: Local anesthetics systemic toxicity
MedDRA: Medical Dictionary for Drug Regulatory Activities
MetHb: Methemoglobinemia
SOCs: System Organ Classes
