# Supplementary material for: Local anesthetics systemic toxicity in children: analysis of the French pharmacovigilance database
Source: BMC Pediatr. 2023 Jun 24;23:321. doi: 10.1186/s12887-023-04126-7 (PMC10290397; doi:10.1186/s12887-023-04126-7)
Supplement: Supplementary file 1 — Additional file 1: Supplementary Table 1. Clinical presentation and univariate statistical analysis of life-threatening and non-life-threatening cases. [file 12887_2023_4126_MOESM1_ESM.docx]

**Supplementary Table 1: Clinical presentation and univariate statistical analysis of life-threatening and non-life-threatening cases.**

|  | Life-Threatening cases | Non-Life-Threatening cases | Overall | p-value^a^ |
| --- | --- | --- | --- | --- |
| Sample size, n (%) | 23 (35.9) | 41 (64.1) | 64 (100) |  |
| Sex, n (%) |  |  |  | 0.65 |
| Boy | 15 (65.2) | 23 (56.1) | 38 (59.4) |  |
| Age, n (%) |  |  |  | 0.15 |
| Newborn (0 - 28days) | 5 (21.7) | 2 (4.9) | 7 (10.9) |  |
| Infant (29 days - 2years) | 8 (34.8) | 11 (26.8) | 19 (29.7) |  |
| Child (2 - 11years) | 6 (26.1) | 17 (41.5) | 23 (35.9) |  |
| Adolescent (12 - 18years) | 4 (17.4) | 11 (26.8) | 15 (23.4) |  |
| Type of local anesthetics, n (%) |  |  |  | 0.0008 |
| Lidocaine | 15 (65.2) | 15 (36.6) | 30 (46.9) |  |
| Lidocaine +Prilocaine | - | 14 (34.1) | 14 (21.9) |  |
| Ropivacaine | 6 (26.1) | 3 (7.3) | 9 (14.1) |  |
| Mepivacaine | 1 (4.3) | 3 (7.3) | 4 (6.2) |  |
| Lidocaine + Bupivacaine | - | 4 (9.8) | 4 (6.2) |  |
| Bupivacaine | 1 (4.3) | 2 (4.9) | 3 (4.7) |  |
| Context of use, n (%) |  |  |  | 0.016 |
| General and orthopaedic surgery | 9 (39.1) | 7 (17.1) | 16 (25) |  |
| Small act of surgery or diagnosis | 4 (17.4) | 8 (19.5) | 12 (18.8) |  |
| Posthectomy | 6 (26.1) | 4 (9.8) | 10 (15.6) |  |
| Dental surgery | 2 (8.7) | 6 (14.6) | 8 (12.5) |  |
| Cutaneous excision | - | 8 (19.5) | 8 (12.5) |  |
| Vaccination | - | 6 (14.6) | 6 (9.4) |  |
| Tonsillectomy | 1 (4.3) | 2 (4.9) | 3 (4.7) |  |
| Involuntary ingestion | 1 (4.3) | - | 1 (1.6) |  |
| Route of administration, n (%) |  |  |  | 0.0041 |
| Local-regional and nerve block | 17 (77.3) | 15 (36.6) | 32 (50.8) |  |
| Topical | 1 (4.5) | 16 (39) | 17 (27) |  |
| Epidural | 3 (13.6) | 5 (12.2) | 8 (12.7) |  |
| Dental | 1 (4.5) | 5 (12.2) | 6 (9.5) |  |
| Missing^b^ | 1 | 0 | 1 |  |
| Settings at adverse reaction onset, n (%) |  |  |  | 0.46 |
| Hospital | 11 (47.8) | 18 (43.9) | 29 (45.3) |  |
| General practitioner's office | 4 (17.4) | 13 (31.7) | 17 (26.6) |  |
| Private clinic | 5 (21.7) | 3 (7.3) | 8 (12.5) |  |
| Dentist's office | 2 (8.7) | 4 (9.8) | 6 (9.4) |  |
| Home | 1 (4.3) | 3 (7.3) | 4 (6.2) |  |
| Compliance with recommended doses, n (%) |  |  |  | 0.78 |
| Dose below the maximum recommended dose | 9 (45.0) | 15 (37.5) | 24 (40.0) |  |
| Above maximum recommended doses | 6 (30.0) | 16 (40.0) | 22 (36.7) |  |
| Dose used not known/no recommendation | 5 (25.0) | 9 (22.5) | 14 (23.3) |  |
| Missing^c^ | 3 | 1 | 4 |  |

a: Quantitative variables were compared in non-parametric Wilcoxon tests and proportions were compared in Fisher’s exact tests or chi-squared tests, as appropriate

b: One case of involuntary ingestion not included

c: LAST cases (n=4) resulting from mistakes not included
