# Supplementary material for: Local anesthetics systemic toxicity in children: analysis of the French pharmacovigilance database
Source: BMC Pediatr. 2023 Jun 24;23:321. doi: 10.1186/s12887-023-04126-7 (PMC10290397; doi:10.1186/s12887-023-04126-7)
Supplement: Supplementary file 2 — Additional file 2: Supplementary Table 2. Description of cases reporting plasma level of local anesthetic. [file 12887_2023_4126_MOESM2_ESM.docx]

**Supplementary Table 2: Description of cases reporting plasma level of local anesthetic.**

|  | Age | Weight  (kg) | Context of use | Type of LA | Route of administration | Dose  (mg/kg) | Time to onset  (min) | Therapeutic management | Sampling delay | Plasma level | Toxic treshold | Adverse reactions |
| --- | --- | --- | --- | --- | --- | --- | --- | --- | --- | --- | --- | --- |
| Case 1 | 18 years | - | Tonsillectomy | Lidocaine | local-regional and nerve block |  | Few minutes | Simple monitoring | - | 2.3 mg/L | > 5 mg/L | Malaise, Loss of consciousness, Convulsion |
| Case 2 | 4 months | 6 | Posthectomy | Lidocaine | local-regional and nerve block | 16 | 20 | Symptomatic  Lipid emulsion | Two half-lives | 4 mg/L | > 5 mg/L | Seizures, Tachycardia, Hypoxia, Cardiorespiratory arrest |
| Case 3 | 4 months | 6.25 | Posthectomy | Lidocaine | local-regional and nerve block | 1.6 | 1 | Symptomatic | 24h | 1.8 mg/L | > 5 mg/L | Convulsion, Bradycardia, Cyanosis |
| Case 4 | 4.5 years | 20 | General and orthopaedic surgery | Lidocaine | local-regional and nerve block | 120 | 0 | Symptomatic | Few hours | 1.4 mg/L | > 5 mg/L | Sleepiness, Bradycardia, Hypotension, Cardiorespiratory arrest |
| Case 5 | 16 months | 13 | Small act of surgery or diagnosis | Lidocaine | Topical | 10.8 | 60 | Symptomatic | at the time of the convulsion | 8 ng/mL | > 5 mg/L | Convulsion |
| Case 6 | 1 months | 3 | General and orthopaedic surgery | Lidocaine | local-regional and nerve block | 10 | - | Symptomatic | 24h | 1.3 mg/L | > 5 mg/L | Seizures, Coma |
| Case 7 | 14 years | 57 | Dental Surgery | Mepivacaine | Dental | 1.3 | 3 | Unknown | 1h | 0.946 µg/mL | > 5 mg/L | Convulsion |
| Case 8 | 3 years | Unknown | General and orthopaedic surgery | Ropivacaine | local-regional and nerve block | - | 5 | Symptomatic  Lipid emulsion | 1.5h | 1.74 mg/L | > 4 mg/L | Seizures |
| Case 9 | 17 years | 45 | General and orthopaedic surgery | Ropivacaine | local-regional and nerve block | 0.9 | 300 | Symptomatic | 6h 9h | 0.19 mg/L 0.09 mg/L | > 4 mg/L | Conduction disorders, Tachycardia |
| Case 10 | 1 months | 3.76 | General and orthopaedic surgery | Ropivacaine | Epidural | 1.1 mg/kg/h | 540 | Symptomatic | 21h after the start of the infusion (14h after stopping the infusion) | 3.5 mg/L | > 4 mg/L | Seizures, Bradycardia, Hypoxia, Cardiorespiratory arrest, Cyanosis, Hypotonia |
| Case 11 | 14 years | 31 | General and orthopaedic surgery | Ropivacaine | Epidural | 0.4 mg/kg/h | 2880 | Symptomatic | 2h after stopping the infusion | 1.956 mg/ml | > 4 mg/L | Dysarthria, Confusion, Sleepiness, Abnormal movements, Bradycardia, Hypoxia |
| Case 12 | 5 years | Unknown | Cutaneous excision | Lidocaine + prilocaine Cream 5% | Topical | - | 60 | Simple monitoring | 1.5h | lidocainemia : 5.5 mg/L prilocainemia:  2 mg/L |  | Confusion, Convulsion, Cyanosis, Methemoglobinemia |
| Case 13 | 5 years | Unknown | Cutaneous excision | Lidocaine + prilocaine Cream 5% | Topical | - | 270 | Simple monitoring | 7h | lidocainemia : 0.7 mg/L | > 5 mg/L | Convulsion |
| Case 14 | 2 months | 5.7 | Vaccination | Lidocaine + prilocaine Patch 5% | Topical | - | 660 | Symptomatic | 3h after removal of the patch (application time: 11h) | lidocainemia: 0.54 mg/ml | > 5 mg/L | Cyanosis, Methemoglobinemia |
| Case 15 | 7 weeks | 4.8 | Vaccination | Lidocaine + prilocaine Patch 5% | Topical | - | 960 | Symptomatic | 2h | lidocainemia: 0.612 mg/L prilocainemia : 0.2 mg/L | lidocainemia :   > 5 mg/L  prilocainemia :  > 5 mg/L | Cyanosis, Methemoglobinemia |

LA: local anesthetic
