# Supplementary material for: Local anesthetics systemic toxicity in children: analysis of the French pharmacovigilance database
Source: BMC Pediatr. 2023 Jun 24;23:321. doi: 10.1186/s12887-023-04126-7 (PMC10290397; doi:10.1186/s12887-023-04126-7)
Supplement: Supplementary file 3 — Additional file 3: Supplementary Table 3. Description of adverse effects and management of life-threatening and non-life-threatening cases. [file 12887_2023_4126_MOESM3_ESM.docx]

**Supplementary Table 3: Description of adverse effects and management of life-threatening and non-life-threatening cases.**

|  | Life Threatening cases | Non-life-threatening cases | Overall |
| --- | --- | --- | --- |
| Sample size, n (%) | 23 (35.9) | 41 (64.1) | 64 (100) |
| Seriousness, n (%) |  |  |  |
| Yes | 22 (95.7) | 35 (85.4) | 57 (89.1) |
| Adverse effects, n (%) |  |  |  |
| Neurological | 31 (46.3) | 59 (64.1) | 90 (56.6) |
| *Unique convulsion* | *1 (1.5)* | *19 (20.7)* | *20 (12.6)* |
| *Seizures* | *18 (26.9)* | *-* | *18 (11.3)* |
| *Malaise* | *2 (3)* | *11 (12)* | *13 (8.2)* |
| *Sleepiness* | *3 (4.5)* | *9 (9.8)* | *12 (7.5)* |
| *Hypotonia* | *3 (4.5)* | *5 (5.4)* | *8 (5)* |
| *Abnormal movements* | *1 (1.5)* | *4 (4.3)* | *5 (3.1)* |
| *Loss of consciousness* | *-* | *3 (3.3)* | *3 (1.9)* |
| *Coma* | *2 (3)* | *1 (1.1)* | *3 (1.9)* |
| *Cries* | *1 (1.5)* | *1 (1.1)* | *2 (1.3)* |
| *Other* |  | *2 (2.2)^a^* | *2 (1.2)* |
| *Dysarthria* | *-* | *2 (2.2)* | *2 (1.3)* |
| *Confusions* | *-* | *2 (2.2)* | *2 (1.3)* |
| Cardiorespiratory, n (%) | 36 (53.7) | 26 (28.2) | 62 (39) |
| *Cyanosis* | *6 (9)* | *8 (8.7)* | *14 (8.8)* |
| *Bradycardia* | *8 (12)* | *5 (5.4)* | *13 (8.2)* |
| *Hypoxia* | *8 (12)* | *4 (4.3)* | *12 (7.5)* |
| *Cardiorespiratory arrest* | *6 (9)* | *-* | *6 (3.8)* |
| *Conduction disorders* | *-* | *5 (5.4)* | *5 (3.1)* |
| *Tachycardia* | *3 (4.5)* | *2 (2.2)* | *5 (3.1)* |
| *Hypotension* | *2 (3)* | *1 (1.1)* | *3 (1.9)* |
| *Respiratory distress* | *2 (3)* | *1 (1.1)* | *3 (1.9)* |
| *Cardiac arrest* | *1 (1.5)* | *-* | *1 (0.6)* |
| Methemoglobinemia | - | 7 (7.6) | 7 (4.4) |
| Time to onset, median [IQR] in min | 20 [5-30] | 30 [5-150] | 20 [5-112.5] |
| < 10 min, n (%) | 6 (26.1) | 12 (29.3) | 18 (28.1) |
| 10-60 min, n (%) | 9 (39.1) | 13 (31.7) | 22 (34.4) |
| 1-12 h, n (%) | 4 (17.4) | 7 (17.1) | 11 (17.2) |
| >12 h, n (%) | - | 3 (7.3) | 3 (4.7) |
| Unknown, n (%) | 4 (17.4) | 6 (14.6) | 10 (15.6) |
| Place of care, n (%) |  |  |  |
| Conventional hospitalization | 6 (26.1) | 27 (65.9) | 33 (51.6) |
| Intensive care unit | 12 (52.2) | 6 (14.6) | 18 (28.1) |
| Hospitalization unspecified | 3 (13) | 6 14.6) | 9 (14.1) |
| No hospitalization | 2 (8.7) | 2 (4.9) | 4 (6.25) |
| Therapeutic Management, n (%) |  |  |  |
| Symptomatic treatment | 18 (78.3) | 16 (39.0) | 34 (53.1) |
| Simple monitoring | 1 (4.4) | 15 (36.6) | 16 (25 ) |
| Unknown | 1 (4.4) | 5 (12.2) | 6 (9.4) |
| Lipid emulsion | 3 (13.0) | 1 (2.4) | 4 (6.3) |
| No treatment | - | 4 (9.8) | 4 (6.3) |
| Final outcome, n (%) |  |  |  |
| Recovery | 21 (91.3) | 41 (100) | 62 (96.9) |
| Death | 2 (8.7) | - | 2 (3.1) |

IQR: interquartile range

a: Vertigo and memory disorders
