# Supplementary material for: Local anesthetics systemic toxicity in children: analysis of the French pharmacovigilance database
Source: BMC Pediatr. 2023 Jun 24;23:321. doi: 10.1186/s12887-023-04126-7 (PMC10290397; doi:10.1186/s12887-023-04126-7)
Supplement: Supplementary file 4 — Additional file 4: Supplementary Figure 1. Number of reported LAST cases by molecule. [file 12887_2023_4126_MOESM4_ESM.docx]

**Supplementary Figure 1: Number of reported LAST cases by molecule.**
